# Intralesional methotrexate versus 5-flurouracil in the treatment of keratoacanthoma

**DOI:** 10.1007/s00403-024-03139-1

**Published:** 2024-06-15

**Authors:** Ahmad Nofal, Rania Alakad, Reham Wahid, Heba Allah Mohamed Hoseiny

**Affiliations:** 1 Dermatology, Venereology &Andrology Department, Zagazig University Hospitals, Zagazig University, Zagazig, Egypt; 2 Physiology Department, Zagazig University Hospitals, Zagazig University, Zagazig, Egypt

**Keywords:** Keratoacanthoma, Intralesional, Methotrexate, 5-flurouracil

## Abstract

**Background:**

Keratoacanthoma (KA) is a benign neoplasm that affects mainly photodamaged skin. It is locally destructive and may rarely spread. Surgery is not always suitable and usually disfiguring. Thus, non-operative modalities represent good alternatives.

**Objective:**

To assess and compare the efficacy of intralesional methotrexate (MTX) and 5-flurouracil (5-FU) in the treatment of KA.

**Patients and methods:**

Randomized controlled trial included 20 patients with biopsy proven KA divided into 2 equal groups; group (A) received intralesional MTX, 25 mg/ml and group (B) received intralesional 5-FU, 50 mg/ml every 2 weeks till complete clearance or for a maximum 5 sessions.

**Results:**

In the MTX group, complete clearance was observed in 7 patients (70%) compared to 8 patients (80%) in the 5- FU group with no statistically significant difference. However, the median number of injections needed to achieve complete response in the MTX group was 3 sessions versus only 2 sessions in the 5-FU group.

**Limitations:**

the small sample size due to the relatively low incidence of KAs in our population.

**Conclusion:**

Intralesional therapy is a good alternative to surgery in selected cases of KA. Both drugs showed comparable efficacy, but 5-FU may give faster results, hence increasing patient satisfaction and compliance.

## Introduction

Keratoacanthoma (KA) is a type of non-melanoma skin cancer (NMSC) arising from follicular epithelial cells. Sometimes, it is considered a low grade but well differentiated subtype of squamous cell carcinoma (SCC) that may undergo spontaneous regression or rarely transforms to invasive SCC. Despite being less aggressive than other types of SCC, it can grow rapidly into large tumors and cause local tissue destruction. Thus, an early therapeutic intervention is recommended [1].

Although surgical excision is the role in KA, minimally invasive options may be considered as an adjuvant or alternative to surgery in selected cases. These options include topical treatments such as 5-fluorouracil (5-FU) and imiquimod, intralesional therapy, cryotherapy, photodynamic or radiation therapy and ablative lasers [2]. These modalities are suitable for patients with solitary KA, recurrent KA, lesions in sites where surgery may lead to significant scarring or disfigurement and lesions in difficult anatomical locations e.g. nose. They can also be used in patients who refuse surgery and those who aren’t fit for the intervention due to old age or associated comorbidities [3].

Intralesional injection of cytotoxic agents has been successfully used in the treatment of NMSC in previous reports [4–8]. In the treatment of KA, several intralesional agents have been tried e.g. methotrexate (MTX), 5-flurouracil (5-FU), interferon and bleomycin with variable results. Despite their reported efficacy, comparative studies are still lacking and there is no standardized protocol for the appropriate concentration, dose, and interval of injection. The aim of this study is to compare the efficacy and side effects of intralesional methotrexate and 5-FU in the treatment of KA in 20 Egyptian patients.

## Materials and methods

The present study included 20 patients with biopsy proven KA collected from Dermatology outpatient clinics at Zagazig University Hospitals in the period from June 2021 to April 2023. Written informed consent was taken from all the patients included in the study. The study protocol was approved by the institutional review board (IRB), Faculty of Medicine, Zagazig university.

Adult patients of either sex were included. They were divided randomly and equally into 2 groups; group (A) included 10 patients who received intralesional injection of methotrexate 25 mg/ml (0,5 − 1 mL/ lesion) and group (B) included 10 patients who received intralesional injection of 5- FU 50 mg/ml (0.5-1 mL/lesion). In tumors < 2 cm, 0.5 ml of MTX or 5-FU was injected while in tumors > 2 cm, 1 ml of MTX or 5-FU was administered. The tumor was imaginably divided into 2 halves and the drug was equally injected in the center of each half. Sessions were carried out every 2 weeks till clearance of the tumor or for a maximum 5 sessions. Exclusion criteria included pregnant and lactating females, patients < 12 years old, hepatic, renal or blood disorders, patients with known hypersensitivity to either drug components, or those who received any treatment for KA at least 1 month prior to our study.

For possible systemic absorption of MTX, baseline and follow-up monitoring of complete blood count, liver function tests, blood urea nitrogen and creatinine levels were performed. In the 5- FU group, baseline and follow-up monitoring of complete blood count was done. Side effects were reported, and clinical photographs were taken before starting treatment and at each subsequent visit. Two dermatologists blindly assessed the tumor regression clinically. There were 3 grades of treatment response; complete, partial and no response (100%, 50–99% and less than 50% resolution respectively). Monthly follow up was done after complete resolution of the tumor to record any recurrence.

### Statistical analysis

The statistical software SPSS version 23 was used to analyze the data. The data were presented as mean ± standard deviation (SD), range, frequencies, and percentages. χ2, Mann-Whitney and *T* tests were used appropriately, and Spearman rank correlation equation was used to correlate variables. A statistically significant result was defined as *P* < 0.05, and a highly significant result as *P* < 0.001.

## Results

Twenty patients with keratoacanthoma were included in the study; 9 males (45%) and 11 females (55%). They ranged from 32 to 60 years old, with a mean age of 49 ± 8.98 years in the MTX group and 43.3 ± 7.5 years in the 5-FU. Among the participants, 25% were smokers and 15% were diabetics. Chronic sun exposure due to long out-door working hours was reported in 11 patients (55%) while a single patient had xeroderma pigmentosa (pigmented xerodermoid variant) since childhood. No significant difference was observed between the two studied groups regarding the demographic data. No history of systemic immunosuppression e.g. organ transplantation or immunosuppressive therapy. Previous history of NMSC was reported in one patient in each treatment group. The baseline characteristics of the studied patients are demonstrated in (Table 1).

**Table 1:** Basic characteristics, clinical parameters of the studied groups

Demographic Data		Methotrexate Group *N* = 10	5- FU Group *N* = 10	Test value	*P* value
Age		49 ± 8.98	43.3 ± 7.5	1.53	0.141(NS)
Gender	Female	6 (60.0%)	5 (50.0%)	0.202	0.653(NS)
	Male	4 (40.0%)	5 (50.0%)		
smoking	Yes	2 (20.0%)	3(30.0%)	0.267	0.606 (NS)
	No	8(80.0%)	7(70.0%)		
Diabetes	Yes	2(20.0%)	1(10.0%)	0.392	0.531 (NS)
	No	8(80.0%)	9(90.0%)		
Transplant/ immunosuppressives		0 (0.0%)	0 (0.0%)	0.000	1.000 (NS)
History of NMSC		1(10.0%)	1(10.0%)		

NMSC: non melanoma skin cancer, (NS): Non-Significant

**Table 2:** KA Lesion characteristics and treatment response. NMSC: non melanoma skin cancer, (NS): Non-Significant (*P* > 0.05)

			Methotrexate Group *N* = 10	5- FU Group *N* = 10	Test value	*P* value
Lesion site		Head	8 (80.0%)	9 (90.0%)	0.392	0.531 (NS)
		Other sites	2 (20.0%)	1 (10.0%)		
Lesion number / patient			1	1	0.000	1.000 (NS)
Lesion diameter (cm)			3.3 ± 1.94	2.1 ± 0.87	1.77	0.100 (NS)
Injection sessions		1	0 (0.00%)	2(20.0%)	5.067	0.281(NS)
		2	2(20.0%)	4(40.0%)		
		3	3(30.0%)	2(20.0%)		
		4	2(20.0%)	0(0.00%)		
		5	3(30.0%)	2(20.0%)		
Median number of injections needed to achieve complete resolution		1	0(0.00%)	2(20.0%)	4.821	0.185 (NS)
		2	2(20.0%)	4(40.0%)		
		3	3(30.0%)	2(20.0%)		
		4	2(20.0%)	0(0.00%)		
Response	Complete		7(70.0%)	8(80.0%)	0.400	0.819 (NS)
	Partial		2(20.0%)	1(10.0%)		
	No response		1(10.0%)	1(10.0%)		
Recurrence after 6 months follow-up			0(0.00%)	0(0.00%)	0.000	1.000 (NS)

**Fig. 1:**
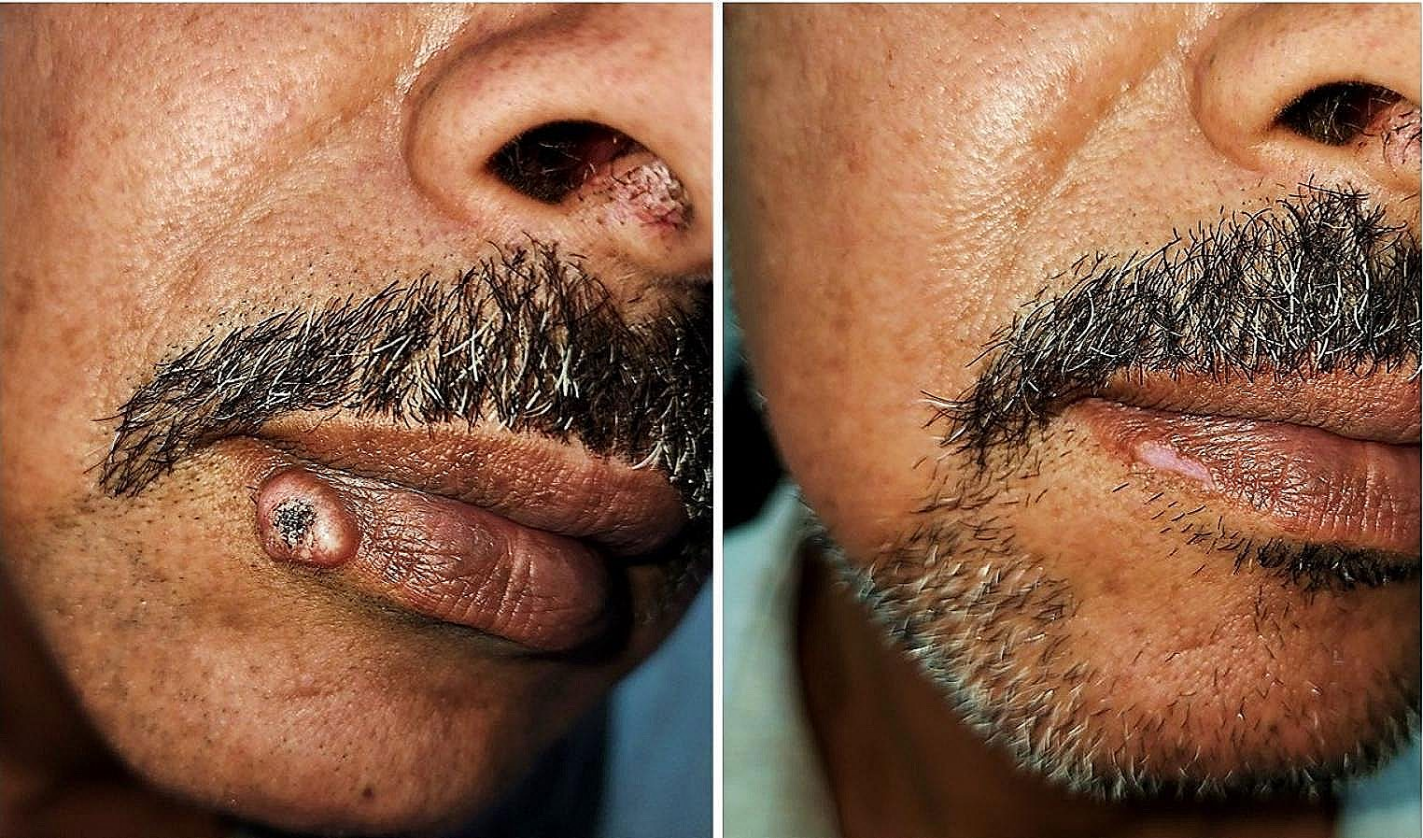
(**A**) keratoacanthoma of the lip before treatment. (**B**) 1 month after 3 sessions of intralesional 5-FU

**Fig. 2:**
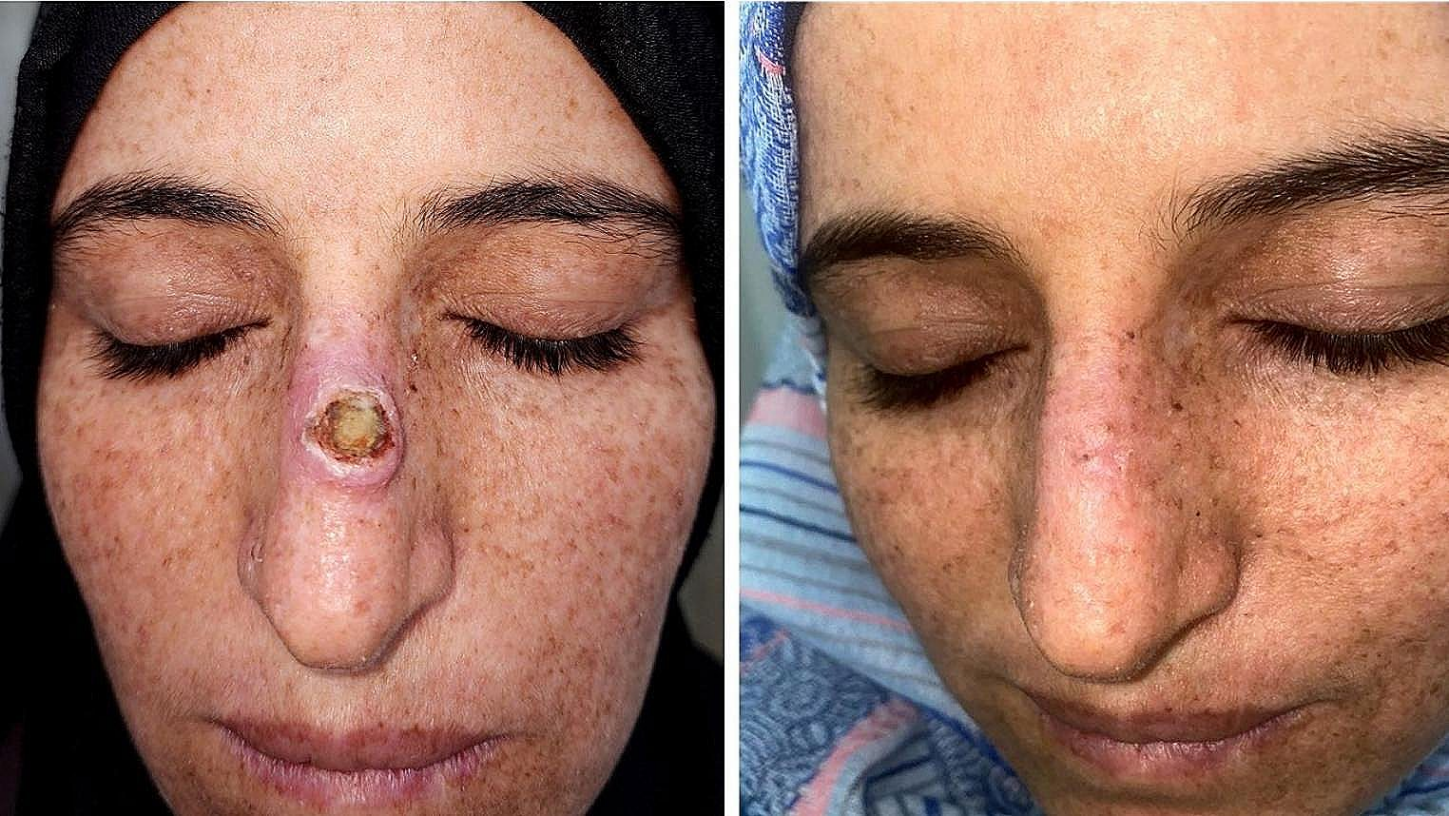
(**A**) large keratoacanthoma of the nose in a xerodermoid female patient before treatment. (**B**) 2 months after 5 sessions of intralesional methotrexate

Regarding the lesion characteristics (Table 2), the head was the most commonly predilected site (85%). Lesions in other sites e.g. hands, feet and arms were only encountered in 15% of the patients. All our subjects had solitary tumors and the diameter of KA ranged from 1 to 4 cm. All of our patients completed the study. In the 5- FU group, 8 patients (80%) showed complete resolution (Fig. 1), one patient (10%) showed partial response and a single patient (10%) showed no change of the tumor size. In the MTX group, complete clearance was observed in 7 patients (70%), (Fig. 2), partial response in 2 patients (20%) and no response in a single patient (10%). At the end of treatment sessions, there was no statistically significant difference between the two groups.

The median number of injections needed to achieve complete response in the MTX group was 3 sessions (5 patients showed complete involution of the tumor after 3 sessions). Meanwhile, the median number in the 5-FU group was 2 sessions (6 patients achieved complete involution of the tumor after 2 sessions). All patients reported tolerable pain during injection. No other adverse effects were recorded. No recurrence was reported during follow-up period.by the patients who showed complete clearance of their tumors.

## Discussion

Keratoacanthoma is a common epidermal neoplasm that may show, in some instances, spontaneous involution, however this is not guaranteed in all cases. It may continue to enlarge and destroy surrounding tissues causing disfigurement or disability or even transforms into SCC in few cases [4]. Thus, early management is recommended to improve the overall functional and cosmetic outcome. Although surgery is the role in the treatment of KA, it is not always practical. Non-operative options are now gaining attention and the selection of the appropriate modality should be case-dependent. These options have high cure rate, better cosmetic results and less serious side effects compared to surgical excision [3].

Considering risk factors for KA, sun damage “like other NMSCs” is the most important factor, especially in fair complexions [9]. Most of our Egyptian population are of skin phototype IV who are less likely to develop NMSC. Genetic disorders with defective DNA repair e.g. Xeroderma pigmentosum (XP) increases the risk of skin cancers at younger age, including KA, squamous cell carcinoma, and melanoma [9]. The incidence of XP is relatively high in Egypt, most probably due to high rate of positive consanguinity [10]. In addition, prolonged sun exposure due to agricultural work represent another predisposing factor to develop NMSC, even with darker complexions. Due to socio-economic factors in our country, patients with skin cancer often present at an advanced stage, making treatment difficult with more incidence of tissue destruction and invasion.

Non-surgical modalities include topical treatment which can be suitable for patients who have a small single lesion and refuse surgery. Both topical 5-FU and imiquimod 5% showed good and comparable efficacy, although topical 5-FU achieved faster results [11, 12]. Systemic drugs can also be used as a possible non-invasive option, especially in case of eruptive widespread lesions. Oral acitretin has been used alone [13] or in combination with either topical imiquimod [14] or intralesional MTX [15]. Bieber et al. reported good response of 3 cases with KAs to subcutaneous injection of MTX after failure of previous treatment with acitretin, cyclosporin and topical 5-FU [16]. Nofal et al. [17] reported a rare case of generalized eruptive keratoacanthoma that showed poor response to acitretin plus methotrexate for 3 months but responded well to cyclophosphamide pulse therapy 1 gm/month for 6 months. Due to systemic side effects, the use of systemic drugs is limited to resistant cases or disseminated tumors.

Intralesional injection of cytotoxic drugs is an effective non-surgical treatment option. Unlike topical treatment, intralesional injection can be used in larger KAs when surgery is not feasible due to patient or lesion-related factors. Intralesional injection allows maximum delivery of the drugs to the tumor without the significant toxicity related to systemic drugs [4]. As larger tumors can be associated with large tissue defect after surgical removal, intralesional injection can be used prior to surgery to minimize the tumor size, reduce the scarring, and improve cosmetic results [2].

Multiple cytotoxic drugs have been tested for the treatment of KA with variable results. Chitwood et al. [2] studied the efficacy and cost of intralesional agents and found the cure rate to be 91% for MTX compared to 98% for 5-FU, 83% for IFN α-2a and 100% for both bleomycin and IFN α-2b.

Methotrexate is the most used intralesional agent followed by 5-FU. Therefore, we decided to compare the efficacy and safety of both drugs in the treatment of KA. Regarding the injection technique, the size of the tumor is the most determining factor. Small lesions are better injected singly into the center till blanching while larger lesions may require multiple injections into several points to ensure the best distribution of the drug. It is better to avoid injecting below the lesion to minimize drug leakage and systemic absorption [7, 8].

Methotrexate is a folic acid analogue that inhibits DNA synthesis in rapidly growing tumors. It has been used in several studies with a dose ranging from 12.5 to 25 mg using a 30-gauge needle every 1–2 weeks. The treatment protocol of intralesional injection in KA is not yet standardized. For better outcome, we decided to use higher concentration of MTX (25 mg/ ml) in a volume of 0.5 ml in smaller lesions and not exceeding 1 ml in larger lesions. The injection was administered every 2 weeks.

After 5 sessions, 70% of our patients showed complete regression of the tumor, while 20% showed partial response and a single patient didn’t respond to treatment. The clearance rate in our study was less than previous reports by Annest et al. [7] and Yoo et al. [18] who reported 92% and 91% clearance rates respectively. This could be related to late onset of treatment in our patients. As we previously mentioned, our patients usually seek medical advice late in the course of the disease. Yoo et al. [18] suggested that intralesional chemotherapy may be a reasonable option only in the rapid growth phase of tumor. It is most effective when started early in lesions not exceeding 3 months duration. The number of injections required to achieve complete response after MTX injection ranges from 2 to 7 sessions [7]. In our study, the median number of MTX injections needed to achieve complete response was 3.

Intralesional injection is most helpful in the treatment of tumors in difficult-to-treat anatomical sites e.g. nose as reconstruction of post-surgical defect in this site can lead to cosmetic disfigurement. In our study, complete resolution of a large KA on the nose of a XP patient was achieved after intralesional MTX injection as demonstrated in (Fig. 2).

The patients who showed regression of their tumors should continue further treatment sessions with intralesional MTX till complete resolution. However, the patient that showed no response to intralesional injection was referred to surgery to avoid treatment delay and progression of the tumor. Laboratory Follow-up is mandatory with intralesional MTX as systemic absorption is a possible side effect, however it wasn’t reported in our study. Annest et al. [7] reported pancytopenia in two of his studied cases due to chronic renal failure.

5-Flurouracil as antimetabolite chemotherapeutic agent, acts through inhibition of thymidylate synthase enzyme responsible for thymidine synthesis thus interfering with DNA replication and arresting tumor growth [19]. Intralesional 5-FU achieved remarkable results in most of the reported literature with efficacy up to 90% [2, 4, 5, 8, 20]. In a review published by Kirby et al., 98.5% of KA lesions injected with 5-FU showed complete regression.

5-Flurouracil has been used in a dose ranging from 15 to 50 mg/ml. Lower doses were recommended in some reports to decrease the treatment-associated pain [20]. In our study, 5-FU was injected in a dose of 50 mg/ml, a volume ranging from 0.5 to 1 ml according to the size of the tumor. Although we used the higher concentration of 5-FU, the pain was tolerated in all patients. After 5 sessions, 80% of the patients showed complete regression of the lesions. The rate of clearance is higher than the MTX group, however no statistically significant difference was found between the two groups.

Despite similar efficacy, 5-FU required fewer number of injections to achieve complete response. After 2 injections, 6 patients showed complete clearance of their lesions in the 5-FU group compared to only 2 patients in the MTX group. That was consistent with the results of a systematic review conducted by Seger et al. [3] which included 15 & 7 articles on MTX and 5-FU respectively. They reported that MTX achieved 92% clearance rate over 4.6 weeks compared to 96% clearance rate by 5-FU over a period of 3.7 weeks. Rapid regression of the tumor and less frequent sessions is an advantage of 5-FU injection as it is associated with better patient adherence, especially that a decision of surgical referral should be made quickly. It is also associated with higher patient satisfaction due to less painful sessions.

As regard the side effects, slight pain and discomfort occurred at the injection site but no significant difference was found between the 2 groups. Kirby et al. [8] claimed that 5-FU can lead to marked irritation, hypopigmentation and scarring at the injection site, especially when used at high concentrations. Nevertheless, we did not experience any of these adverse effects during the study.

None of our patients developed any recurrence during the follow up period which is consistent with that reported in literature. In the case series conducted by Aubut et al. [21]; the follow up period for the cases treated with intralesional MTX ranged from 1 to 24 months with a mean of 3 months. Although no recurrence detected, they recommended longer follow up with regular visits for early detection of any recurrence. Also, Yoo et al. [18] reported no recurrence during the follow up period of 2–84 months with a mean of 18.3 months following intralesional MTX therapy. Considering intralesional 5-FU therapy; the mean follow up period in the reported studies was 21.7 by Marka et al. [20] and over 12 months by Odom et al. [22] with no recurrence at all.

## Limitations

The main limitation of our study was the small sample size which is attributed to the relatively low incidence of KAs in our population. Furthermore, different concentrations of the drugs should be investigated in future studies to achieve a standardized dose of injection. A longer follow-up period is also recommended as tumor recurrence is the main concern for both clinicians and patients. To conclude, intralesional injection of cytotoxic drugs is an effective alternative to surgery in selected cases of KA. Both methotrexate and 5-FU are highly efficient and relatively cheap intralesional drugs. Fewer sessions were required for complete involution of the tumor after 5-FU injection which can be associated with better patient compliance and satisfaction.

## Data Availability

No datasets were generated or analysed during the current study.
